# Assessment of the antileishmanial activity of diallyl sulfide combined with meglumine antimoniate on *Leishmania major*: Molecular docking, *in vitro*, and animal model

**DOI:** 10.1371/journal.pone.0307537

**Published:** 2024-08-30

**Authors:** Farzaneh Zarrinkar, Iraj Sharifi, Ehsan Salarkia, Alireza Keyhani, Zahra Babaei, Ali Khamesipour, Maryam Hakimi Parizi, Elaheh Molaakbari, Fatemeh Sharifi, Shahriar Dabiri, Mehdi Bamorovat

**Affiliations:** 1 Leishmaniasis Research Center, Kerman University of Medical Sciences, Kerman, Iran; 2 Center for Research and Training in Skin Diseases and Leprosy, Tehran University of Medical Sciences, Tehran, Iran; 3 Research Center of Tropical and Infectious Diseases, Kerman University of Medical Sciences, Kerman, Iran; 4 Afzalipour School of Medicine and Pathology and Stem Cells Research Center, Kerman University of Medical Sciences, Kerman, Iran; HNBGU: Hemvati Nandan Bahuguna Garhwal University, INDIA

## Abstract

Currently, no safe vaccine against leishmaniasis is available. So far, different control strategies against numerous reservoir hosts and biological vectors have not been environment-friendly and feasible. Hence, employing medicinal components and conventional drugs could be a promising approach to developing novel therapeutic alternatives. This study aimed to explore diallyl sulfide (DAS), a dynamic constituent of garlic, alone and in a mixture with meglumine antimoniate (MAT as standard drug) using *in vitro* and animal model experiments against *Leishmania major* stages. The binding affinity of DAS and four major defense elements of the immune system (iNOS, IFN-ɣ, IL-12, and TNF-α) was used to predict the predominant binding mode for molecular docking configurations. Herein, we conducted a broad range of experiments to monitor and assess DAS and MAT potential treatment outcomes. DAS, combined with MAT, displayed no cytotoxicity and employed a powerful anti-leishmanial activity, notably against the clinical stage. The function mechanism involved immunomodulation through the induction of Th1 cytokine phenotypes, triggering a high apoptotic profile, reactive oxygen species (ROS) production, and antioxidant enzymes. This combination significantly decreased cutaneous lesion diameter and parasite load in BALB/c mice. The histopathological findings performed the infiltration of inflammatory cells associated with T-lymphocytes, particularly CD4+ phenotypes, as determined by biochemical markers in alleviating the amastigote stage and improving the pathological changes in *L*. *major* infected BALB/c mice. Therefore, DAS and MAT deserve further advanced therapeutic development and should be considered as possible candidates for treating volunteer cases with cutaneous leishmaniasis in designing an upcoming clinical trial.

## Introduction

Leishmaniasis is an overlooked protozoan parasitic disease consisting of different complex clinical presentations induced by various species of the *Leishmania* genus. Three traditional demonstrations of leishmaniasis are visceral, mucocutaneous, and cutaneous (CL) [1–3]. Leishmanias is a main health concern in 101 nations in the endemic foci of Asia, Latin America, and Africa and is responsible for a considerable burden [4, 5].

*L*. *major* and *L*. *tropica* are the causal parasites of CL exclusively in the Eastern Hemisphere and are widely distributed and affect low and middle-income inhabitants in many countries. Currently, no efficacious and safe vaccines are accessible. Moreover, conventional therapies are associated with poor treatment adherence, parasite resistance, and long duration of application [6, 7]. Such limitations of safe and effective control strategies encourage us to search for novel therapeutic alternatives that are widespread and valuable sources of effective medicinal constituents [8, 9].

Several plant-based ingredients and products are proposed for leishmaniasis therapy [10]. Garlic (*Allium sativum*) has been cultivated by humans since ancient times and received additional attention as a natural dietary agent because of the abundance of its bioactive properties, such as anticancer and antioxidant functions [11, 12]. It has been used in old-style medicine for medical behavior such as coronary heart disease, cancer, and infectious diseases [11, 13, 14].

Different cytokines of pro- and anti-inflammatory compositions play a crucial role in CL infection’s susceptibility/resistance and immunopathology. Clinical outcomes will be determined depending on the amount and quality of the Leishmania antigen. T helper1 (Th1) subset, including interleukin 12 (IL-12), gamma interferon (IFN-γ), and tumor necrosis factor-alpha (TNF-α), are the major factors associated with the development of immune response against *L*. *major* infection. In contrast, the Th2 phenotype facilitates the production of IL- 4, 5, 10, 13 and tumor necrosis factor beta (TNF-β) and is directly involved with the persistence and chronicity of the infection [15].

DAS is a product of allicin and the enzyme alliinase, and this results in the biosynthesis of three extra components: diallyl monosulfide, diallyl trisulfide, and diallyl disulfide [16]. DAS consists of organosulfur complexes that give garlic its taste and odor. These constituents have anti-malignancies and anti-mutagen possessions. The wonderful potential of DAS in repressing microbial infections has been confirmed by its content and behavior on a few foodborne infectious bacteria. By shielding against chemically induced hepatoxic effects, reports have indicated that DAS can suppress various chemically induced cancers.

Moreover, DAS possesses hypoglycemic, antibacterial, antiatherosclerosis, and hypolipidemic properties [17, 18]. This study aimed to use DAS alone and along with conventional drugs (Glucantime^®^ or meglumine antimonate; MAT) on *L*. *major* stages through *in vitro* experimentations and animal models. Before the experimentation, we performed molecular docking on DAS and IL-12, IFN-γ, and TNF-α and induced nitric oxide synthase (iNOS), as the central defensive elements of the control strategy against CL. In the search for potent leishmanicidal therapeutic approaches for treating leishmaniasis, combination therapy has invariably been an important strategy to diminish toxicity, prevent the emergence of the parasite, cure faster, and employ broader-range ingredients with different mechanisms of action [19].

## Material and methods

This study is performed in three sections: *in silico*, *in vitro*, and *in vivo*.

### A) *In silico*

#### Target/receptor preparation

The Th1 proliferation and pro-inflammatory cytokines incorporating iNOS, IL-12, IFN-γ, and TNF-α were selected as targets in this study. At first, the protein targets were downloaded structures usable for this work with PDB, ID: 4NOS, 6E3K, 1F45, and 3ALQ from the RCSB database (https://www.rcsb.org/). Afterward, to prepare models for molecular docking, we extracted the redundant in the PDB format operating the MVD software.

#### Molecular docking (MVD) process

The MVD software with 87% precision was operated for the molecular docking process [20]. At first, the protein structures and their mixtures were planned by employing the “protein preparing” and “prepare molecules” in the MVD component, and then cavities of proteins were identified as relevant positions on receptors for ligand binding. Moreover, 0.30Å Grid firmness, 1500 extreme iteration, and 50 supreme sizes were appointed as docking groundwork. To consider the required affinity with the iNOS, IL-12, IFN-γ, and TNF-α were recorded with the internal electrostatic collaboration, sp2-sp2 rotations, and the internal H-bond connections. Also, in the docking process, 10 sets were run pursued by energy minimization through Nelder-Mead Simplex Minimization. The outcomes were considered utilizing PyMOL and Discovery Studio software, and the best-related composite was selected from each dataset.

#### Absorption, distribution, metabolism, and excretion (ADME) and drug-likeness

In the pharmaceutical signifying server titled SwissADME, calculation procedures were employed to consider the ADME and drug-likeness characterization. SwissADME is a free advantageous basis in the ADMET projection for receiving possessions of recent biochemical compounds (http://www.swissadme.ch/, [21].

### B) *In vitro*

#### Drugs and reagents

Diallyl sulfide (DAS) was purchased from Sigma (Germany) with CAS no. 592-88-1. Meglumine antimoniate was purchased from Sanofi-Aventis, France. Drug dilutions were prepared in distilled water to evaluate serial concentrations of 12.5, 25, 50, 100, and 200 μM. Tetrazolium salt (MTT) and dimethyl sulfoxide (DMSO) were used. The FITC Annexin V Apoptosis Detection Kit with 7-AAD Set was obtained from Elabscience (St. Louis, MO, USA). The permeate probe diacetate 2′,7′-dichlorofluorescein (Sigma) was used. Dulbecco’s Modified Eagle’s Medium (DMEM) and fetal bovine serum (FBS) were provided by Biosera (France). The topical treatment of DAS was applied in gel form (in a ratio of 1:1), which was prepared as a cream-based formulation [22].

#### Parasites and cells

The promastigote stage of *L*. *major* national standard isolate (MRHO/IR/75/ER) was maintained from Afzalipour Medical School, Kerman, Iran at 25°C in RPMI1640 monophasic medium complemented with 10% heat-inactivated FBS, and 1% penicillin/streptomycin. A mouse macrophage cell line (J774-A1) was provided by the Kerman Leishmaniasis Research Center and cultivated in DMEM supplemented with 10% FBS, and 0.5% penicillin/streptomycin (Sigma, Poole, UK) at 37°C with 5% CO2 concentration.

#### Anti-promastigote activity

Drug susceptibility assays were performed using logarithmic phase promastigotes. A total of 90 μl of cultivated promastigotes of *L*. *major* (10^6^ cells/mL) were added to a 96-well microtiter plate. Then, 10 μl of predetermined DAS, MAT, or their combination, as mentioned previously, were added to each well and incubated at 25°C for 72 h. The termination of reactions, ELISA reading assessments, and calculation of the IC50 (50% inhibitory concentration) were conducted in SPSS software by probit test [23]. Each experiment was performed three times. We also estimated the combination index (CI) using the following equation: [CI = (D) / (Dx)1 + (D) / (Dx)2], where (Dx)1 and (Dx)2 represent the concentration of DAS and the tested MAT used in solitary treatments required to reduce the cell count by x%, and (D) is the concentration of DAS in combination with the concentration of MAT (in a ratio 1:1) that collectively reduced the cell count by x%. The CI value quantifiably expresses synergism (CI < 1), additive effect (CI = 1), and antagonism (CI > 1). To determine the synergistic activity of combination therapy, we calculated the theoretical IC50 using the following formula: [theoretical IC50 = (IC50 MAT / 2) + (DAS / 2)].

#### Anti-amastigote activity

For estimation of the leishmanicidal activity against *L*. *major* intra-macrophage amastigotes, 200 μL of murine macrophages (J774-A1 cell line) with 10^7^ cells were cultivated for 24 h in coverslip well of 8-compartment slides (Lab-Tek, Nalge Nunc International NY, USA). Then 200 μL of metacyclic promastigotes were relocated beside the macrophages (10:1 ratio). After 24 h, 40 μL of all concentrations of DAS, MAT, or a combination of them were transferred to cells and maintained for 72 h. Cells were washed with 50 μL PBS to remove free parasites, and subsequently, fixed with methanol and Giemsa stained. The minimum concentration of a substance at which 50% of leishmanial organisms are inhibited (IC50) was obtained by visualizing Leishman bodies (amastigotes) in 100 macrophages under an optical microscope. Each test was done in triplicate. The IC50 values were intended in SPSS by probit test.

#### Cytotoxic effects

The mouse macrophages (5×10^5^) were seeded at a prearranged density of the examined medicines in 96-well ELISA microplates at 37°C for 72 h in 5% CO2 pressure to investigate the toxic effect of DAS, MAT, and their mixture performing MTT assay. The remaining experimental procedures followed the same procedures as mentioned before. Lastly, the MTT colorimetric assay was employed to measure cell mortality. The data were expressed as the proportion of nonviable cells in culture media treated with DAS, MAT, and a combination of them relative to the untreated control macrophages.

#### Th1 and Th2 related cytokines

The quantified expressions of selected Th1 and Th2 cytokines were identified employing quantitative real-time PCR (qRT-PCR) test on mouse macrophages infected with *L*. *major* amastigotes and treated with DAS, MAT, and a combination of them. The entire RNA of samples was isolated using the High Pure RNA Isolation Kit (Roche, Basel, Switzerland). Initially, the concentration of RNA was determined using Thermo Fisher Scientific nanodrop, and then cDNA was synthesized by Roche Synthesis Kit. The remaining qRT-PCR processes were carried out based on the protocol reported elsewhere. In Table 1 the primer’s pattern and control gene sequences are presented. Based on the results of earlier *in vitro* experiments, the full study procedure was carried out [23]. The following equation was used to calculate CT: [ΔCT = CT (target) - CT (reference)]. Moreover, the fold change was considered using the comparative threshold approach (ΔΔCT). To verify the length of the qRT-PCR products, we subjected the PCR amplicons to gel electrophoresis and included images of the resulting products in the S1 Raw images section of the supplementary files. Also, to determine the amount of changes in selected Th1 and Th2 cytokines production, after 72 h of treatment with different concentrations of DAS, MA, and a combination of them, the amount of cytokine synthesis in the supernatant was read by ELISA reader using the commercial kit of Thermo Fisher Mouse Cytokines ELISA Kits using the relevant instructions.

**Table 1:** The specific primers and reference gene sequences.

Template	Forward and reverse sequences (5´-3´)		Product size (bp)
TNF-α	Forward	CAGGCGGTGCCTATGTCTC	161
	Reverse	CGATCACCCCGAAGTTCAGTAG	
IFN-γ	Forward	5-GCCGATGATCTCTCTCAAGTGAT-3	106
	Reverse	5-ACAGCAAGGCGAAAAAGGATG-3	
IL-12	Forward	TGGTTTGCCATCGTTTTGCTG	171
	Reverse	ACAGGTGAGGTTCACTGTTTCT	
IL-10	Forward	CTTACTGACTGGCATGAGGATCA	134
	Reverse	GCAGCTCTAGGAGCATGTGC	
TGF-β	Forward	CCACCTGCAAGACCATCGAC	112
	Reverse	CTGGCGAGCCTTAGTTTGGAC	
iNOS	Forward	ACATCGACCCGTCCACAGTAT	89
	Reverse	CAGAGGGGTAGGCTTGTCTC	
SOD	Forward	TATGGGGACAATACACAAGGCT	75
	Reverse	CGGGCCACCATGTTTCTTAGA	
CAT	Forward	GGAGGCGGGAACCCAATAG	102
	Reverse	GTGTGCCATCTCGTCAGTGAA	
GAPDH	Forward	5-AGGTCGGTGTGAACGGATTTG-3	95
	Reverse	5-GGGGTCGTTGATGGCAACA-3	

#### SOD, CAT, and NO

To measure changes in relative gene expression and average production of superoxide dismutase (SOD), catalase (CAT), and nitric oxide (NO) in *L*. *major* intra-macrophage amastigotes, infected macrophages were treated with various concentrations of DAS, MAT, or both for 72 h. qRT-PCR methods were used to identify the mRNA levels of antioxidant proteins, as described above. To determine SOD, CAT, and NO activities in amastigotes, the commercial kit (SOD assay kit, Calbiochem) was used following the manufacturer’s protocol. After following the kit instructions, the protein density was finally calculated using a spectrophotometric test with an ELISA reader.

#### Reactive Oxygen Species

For measuring the average quantity of *L*. *major* intra-macrophage amastigotes ROS, 10^6^ macrophages were treated with IC50 concentrations of DAS, MAT, or a combination. Following 24, 48, and 72 h, the cells were then loaded with 10 M of a permeate probe diacetate 2′.7′-dichlorofluorescein (Sigma) diluted in DMSO and incubated at 37°C for 25 min in 5% atmospheric CO_2_. The cells were then rinsed with PBS (pH 7.3). The Marshall Scientific BD Bioscience FacsCanto II Flow Cytometer was used to control ROS.

#### Apoptotic profile

Apoptosis of promastigotes was studied by well-designed flow cytometry using the BD Annexin V/ PE Apoptosis Detection Kit- Fisher Scientific). Twelve-well plates of promastigotes (10^6^) were used to treat them with various concentrations of DAS, MAT, or both and kept at 25°C for 72 h. After that, the parasite was rinsed with PBS before being incubated for 15 mins at 25°C in the dark with 5:l of PE-Annexin-V and 7:l of 7-AAD. Lastly, the proportion of the apoptotic parasites was evaluated by Flowjo software.

#### Transmission electron microscopy (TEM)

Ultrastructural alterations were envisaged by TEM. Promastigotes were treated with IC50 concentration of DAS, MAT, or a combination for 72 h. The samples were washed with PBS and fixed in 2.5% glutaraldehyde (0.1 M sodium cacodylate buffer pH 7.4). After the dehydration process in an acetone series, sections were stained with 5% uranyl acetate and lead citrate and inspected in a JEOL JEM 1400 TEM.

### C) *In vivo*

#### BALB/c mice

Thirty female BALB/c mice of age 6 weeks old (20-25 g each) provided by the Pasture Institute of Iran, were housed in pathogen-free animal cages with a 12h light-dark cycle at a temperature of 24±2°C, humidity of 40% ± 5% and were fed the same sterile-grade commercial pellet diet and distilled water and ad libitum. They were randomly separated into seven groups of five each, and kept under suitable controlled physical and nutritional conditions. All animal experimentations were completed according to the Iranian Animal Protection Guide of the National Council for the Control of Animal Experiments. The procedure was agreed upon by the Ethics Committee of the Kerman University of Medical Science (IR.KMU.AH.REC.1399.120) and all efforts were made to minimize suffering. Since the studied mice were daily examined by a veterinarian, if severe behavioral changes such as the signs of approaching death, poor prognosis of quality of life, or specific signs of severe suffering or distress were evident, the animals were euthanized. The research staff participating in this study completed the training course on ethics in research and working with laboratory animals and had sufficient knowledge and expertise in this field.

#### Culture of parasites and infection of animals

Infective promastigotes of *L*. *major* were attained from experimentally infected BALB/c mouse footpad lesions after euthanization, cultivated in complete RPMI1640 medium, and kept at 25°C. The animals were anesthetized with Ketamine (80–100 mg/kg, i.p.) and Xylazine (5-10 mg/kg) before infection. After 5 passages, 2×10^6^ stationary-phase promastigotes were prepared, counted, and inoculated by intradermal route to the right hind footpad of BALB/c mice.

#### Treatment and lesion size assessment

After one month post-infection, the mice’s lesion development was monitored and they were randomly separated into 6 groups (5 animals kept in each box) at the end of the study, all 30 mice were alive and euthanized: I. Untreated control (the mice’s lesions that were not treated), II. DAS (oral), III. DAS (topical gel applied by a cotton swab two times/day), IV. MAT, V. DAS (oral) + MAT, and VI. DAS (topical) + MAT. In groups, III and VI mice received DAS in gel formulation using a cotton swab applicator, twice daily. In groups, V, VI, and IV animals received MAT intramuscularly (IM) alternating between the right and left thigh each day. All treatment schedules were used at a dosage of 20 mg/kg/day and continued for 28 days. The average infected right footpad lesion diameter was measured dorso-ventrally and laterally by a digital Kulis Vernier compared to that of the contra-lateral left hind footpad in different control groups of non-infected BALB/c mice. The lesion caused by the *L*. *major* parasite is not painful, and since the mice did not have any special problems during the study, there was no need to use analgesics or special housing conditions.

#### Parasite load quantification

Twelve weeks after infection, the mice were euthanized and some organs were aseptically removed. All BALB/c mice were euthanized with an i.p. injection of Ketamine (600 mg/kg) and Xylazine (30 mg/kg). The right popliteal lymph nodes were removed under aseptic conditions, and they were homogenized in RMPI1640 medium with 10% inactive FBS. After homogenization 4 × 10^6^ cells were counted and kept at -70°C. The parasite burden was quantified by qPCR assay. Total DNA was extracted by Qiagen extraction kit. Gene expression study was achieved by SYBR Green PCR Master Mix (Applied Biosystems) on the Rotorgene Cycler system (Corbett Research, Cambridge, United Kingdom). GAPDH was used as a housekeeping gene for standardization. Primer template sequences were SODB1 (Forward: 5’-TGGTG-GACATCATCAAGT-3’ and Reverse: 5’-AGAAGAAGTCGTGGTTGTA-3’).

#### Histopathological and immunohistochemical examination

Spleens and lymph nodes from BALB/c mice were embedded in paraffin and preserved in 10% formalin. Hematoxylin and eosin (H&E) were used to stain 5 μm tissue slices. Microscopic examination was meticulously done to find the histopathological variations in the spleen of animals and the parasite load was calculated using the Ridley scoring procedure [24]. A Leica Orthoplan microscope took all the images. For immunohistochemical (IHC) estimation we used an anti-CD4 (MU421-UC; clone 4B12) antibody to investigate the changes in the function of Th1.

#### Statistical analysis

All data were analyzed by SPSS Ver 22.00 (Chicago, IL, USA) accompanied by GraphPad Prism version 8.0 (CA, USA). One-way ANOVA and a Student t-test were used to find any significant association among treatment groups at a *P* <0.05 significant level. The data of the one-way ANOVA test is shown in S1 Dataset. The SPSS probit test was used to get the IC50 values. To determine the differences between the IC50 in promastigotes and intracellular amastigotes, the t-test was used. The selectivity index (SI) is a measure of toxicity added to the following equation: Peritoneal macrophage cells: non-toxic, CC50/IC50 = 1 [25].

## Results

### *A)* In silico

**Fig 1:**
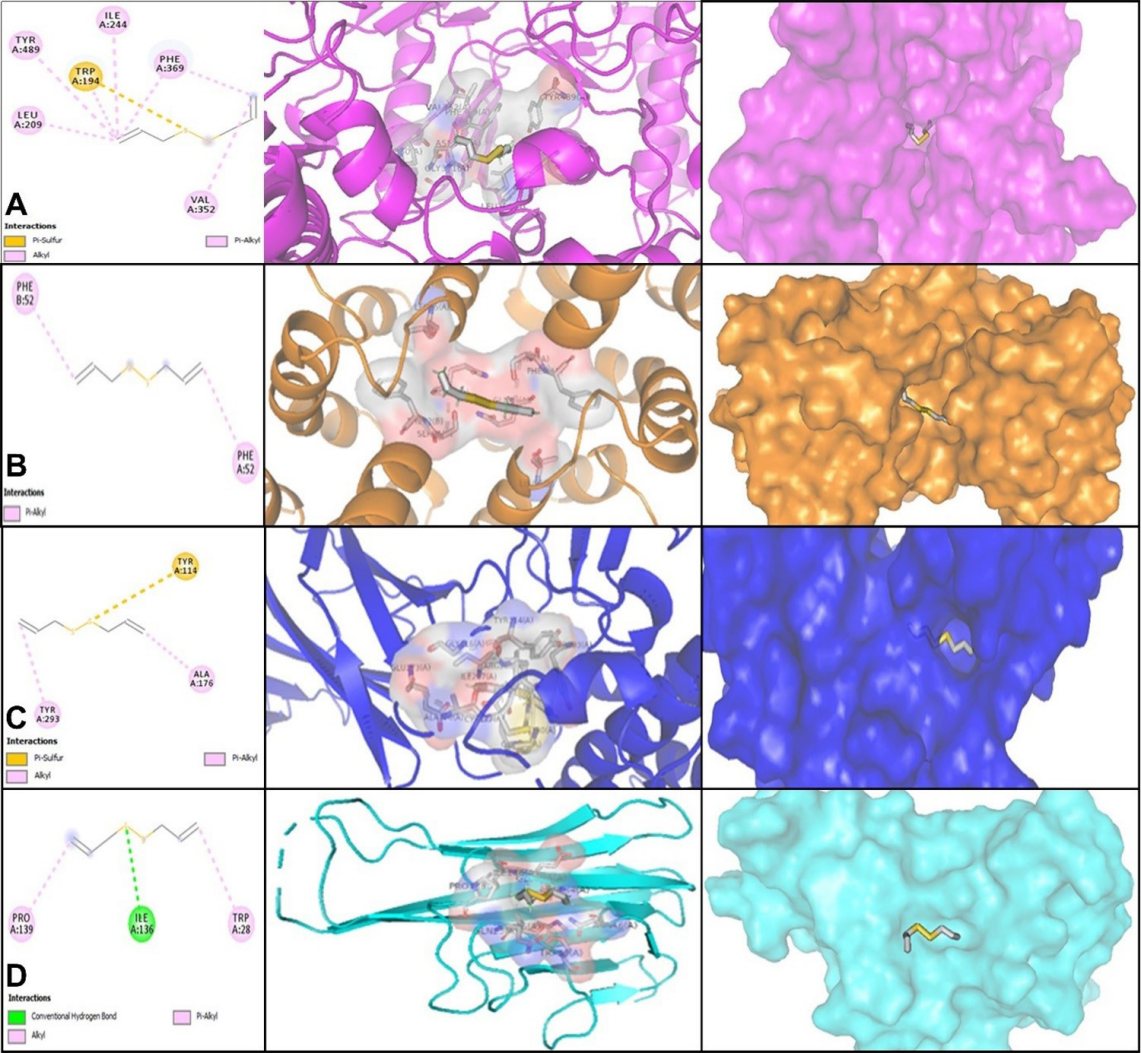
Representation of the best score docking solution of the diallyl sulfide ligand and (A) iNOS, (B) IFN-γ, (C) IL-12, and (D) TNF-α receptor with the selected crystal structure of 4NOS, 6E3K, 1F45, and 3ALQ, respectively, along with the ligand map with various chemical bonds of Discovery Studio.

Configurations of docked DAS with immunomodulatory Th1 cytokines (iNOS, IFN-γ, IL-12, and TNF-α) were considered in the phrase energy. MolDock Scores were indicated by negative energy values, attributing that the binding possibilities of the drugs were impulsive. The docking score of diallyl sulfide was calculated by MVD. Likewise, values of the MolDock Scores of molecular docking of DAS with iNOS, IFN-γ, IL-12, and TNF-α receptors were -67.92, -61.98, -73.06, and -57.01, respectively. *In silico* results and ligand maps of DAS and the 4NOS, 6E3K, 1F45, and 3ALQ targets are illustrated in Fig 1. Diallyl sulfide creates Pi-sulfur, Alkyl, and Pi-Alkyl interactions with amino acid residues (Trp194, Leu 209, Ile244, Val (A)352, Phe(A)369 and Tyr(A)489) of the iNOS receptor (Fig 1A). Moreover, DAS interacts with IFN-γ with the attached site including amino acids remaining similar to Phe52 (A) and Phe52 (B) with Pi-Alkyl interactions (Fig 1B). The resultant data from the molecular docking analysis in Fig 1C provided that DAS forms Pi-sulfur, Alkyl, and Pi-Alkyl connections with amino acids of the IL-12 utilizing Tyr (A) 114, Ala (A) 176, and Tyr (A) 293. Also, DAS stabilized by TNF-α employing conventional hydrogen bonds, Pi-Alkyl, and Alkyl interactions with amino acid deposits; Trp (A) 28, Ile (A) 136, and Pro (A)139 (Fig 1D).

### ADME and drug-likeness predictions

Drug-likeness of the DAS was indicated and established on an earlier-founded idea through Lipinski’s rule [26]. The DAS structure was transformed into the approved molecular input line entrance approach (SMILE). The SwissADME server was asked to analyze *in silico* pharmacokinetics, taking into account a compound’s total polar surface area, the number of rotatable bonds, hydrogen donors, and acceptors. The preference of the compound as a pharmaceutical prospect was decided by a drug score. The significance of the drug outcome and the chance of the drug is regarded as a compound outlook.

To assess orally active medicines, Lipinski’s rule of five is used to establish compound similarity. The hydrogen-bond donors (HBD) should not surpass 5, the hydrogen-bond acceptors (HBA) should not exceed 10, the molecular mass should not exceed 500 Da, the calculated log P should be greater than 5, and the total polar exterior area (TPSA) should not exceed 140, according to the five-parameter instruction. Moreover, Lipinski’s rule of five implies that the medications follow this directive. In this investigation, the SwissADME prediction demonstrated the DAS regarded Lipinski’s rule.

Likewise, the results of ADME. Therefore, the log S value for DAS is -2.9, which reveals adequate solubility in water [27], and the log Kp for cutaneous permeability which signifies the skin absorption of the compound is within -5.63 [28]. Besides gastrointestinal (GI), the glycoprotein P (P-gp) membrane and blood-brain boundary (BBB) infusion designate the absorption and distribution of drugs [29]. These parameters predicted that DAS offered high gastrointestinal (GI) absorption, was not a permeability substrate of glycoprotein (P-gp), and led to BBB permeation. Furthermore, a variety of cytochromes (CYPs) such as CYP1A2, CYP2C19, CYP2C9, CYP2D6, and CYP3A4 administer the metabolism of the drug which they are dynamic for the biotransformation of drug particles [30]. Diallyl sulfide was predicted to not inhibit the cytochromes. Accordingly, based on the ADME prediction study, DAS can be a satisfactory candidate in this investigation (Drug-likeness predictions of diallyl sulfide calculated by SwissADME and ADMET Predictions of diallyl sulfide calculated by SwissADME presented in supplementary file).

### B) *In vitro*

#### Anti-promastigote activity

**Fig 2:**
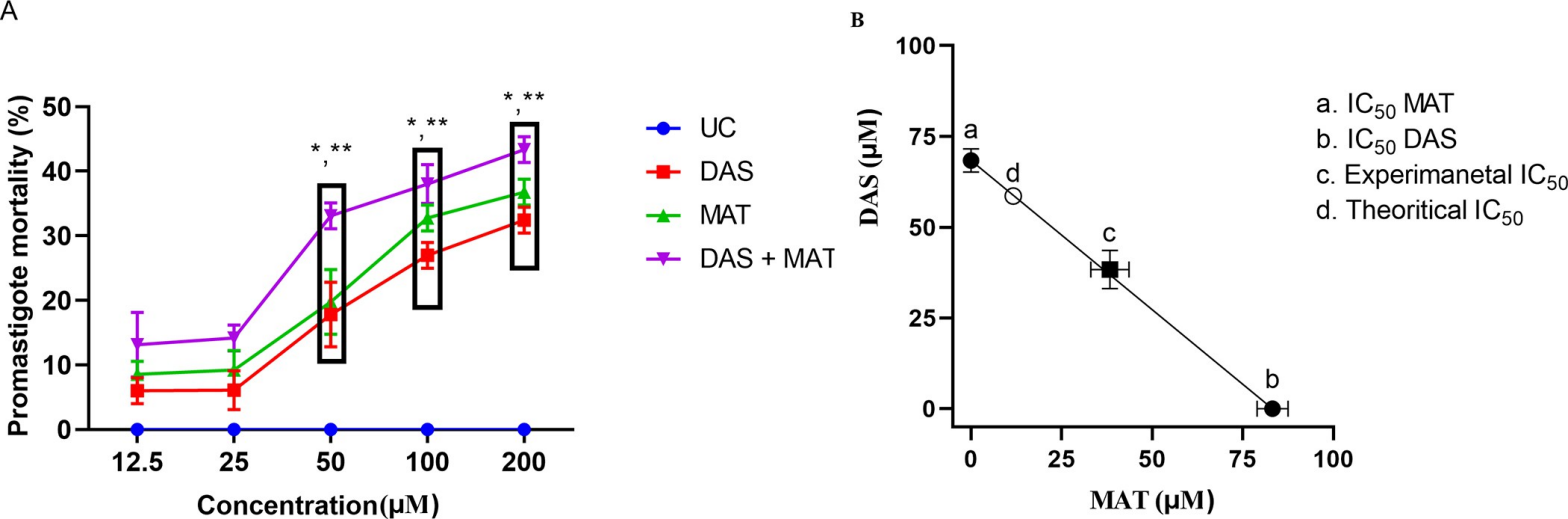
(A) The values of total mean mortality in promastigote of *L*. *major* parasite in different concentrations of diallyl sulfide (DAS), meglumine antimoniate (MAT) alone, and their mixture (DAS+MAT) (*significant difference in comparison to the untreated control group (UC) (* *P* <0.001) (**significant difference between DAS + MAT and DAS and MAT alone (*P* <0.05). (B) The isobologram analysis of the additive effect of the drug combination of diallyl sulfide (DAS) and meglumine antimoniate (MAT). Points a and b display the IC50 value of DAS (68.37 μM) and MAT (83.22 μM), respectively. Theoretical IC50 was 75.79 μM and our experimental IC50 was 38.34 μM. Statistical examination exposed that there was a significant difference between test IC50 and hypothetical IC50 (*P* <0.001).

The average mortality rate of promastigotes at prearranged concentrations of DAS, MAT alone, and their mixtures (in a 1:1 ratio) are shown in Fig 2A. All three treatment modalities showed significant anti-leishmanial activity at diverse concentrations for 72 h of normal incubation relative to the untreated control group, except at 25 μM no detectable effect was noticed. The mortality profile followed a dose-dependent response. The activity was more intense at higher concentrations. The inhibitory effect was superior in combination (*P* <0.001) when compared to each drug alone (*P* <0.01).

#### Anti-amastigote activity

Various concentrations of DAS, MAT alone, and their mixture (in a 1:1 ratio) caused a significant decrease in the overall number of intra-macrophage amastigotes (clinical stage) compared to the untreated control groups (*P* < 0.001). However, they exhibited a greater lethal effect when used in combination. The minimum inhibitory concentration was detected at 200 μM for DAS and MAT alone, while it was 100 μM when used in combination (*P* < 0.0001). Notably, under the same conditions, MAT showed a greater effect than DAS alone. In the combination of DAS with MAT, the lowest IC50 value (38.34 μM) was observed, compared to DAS alone (IC50 = 68.37 μM). Additionally, no toxicity was observed with the drugs against intra-macrophage amastigotes (Table 2). The IC50 value for promastigotes (non-clinical stage) was significantly higher than that of intracellular amastigotes (*P* < 0.001).

**Table 2:** Comparison of the effect of different concentrations of diallyl sulfide (DAS) and meglumine antimoniate (MAT) alone and their combination (DAS+MAT) on the average number of intra-macrophage amastigotes compared to the untreated control group (UC).

Concentration (μM)	DAS		MAT		MAT + DAS	
	Mean±SD	P value	Mean±SD	P value	Mean±SD	P-value
0.0 (UC)^a^	51.44±2.21	NR	51.44±2.21	NR	51.44±2.21	NR*
12.5	43.11±3.21	<0.001	39.86±2.33	<0.001	31.95±3.21	<0.001
25	39.83±3.86	<0.001	31.42±1.28	<0.001	24.34±3.22	<0.001
50	31.86±2.36	<0.001	28.33±3.82	<0.001	17.31±2.33	<0.001
100	19.33±1.56	<0.001	14.32±2.11	<0.001	0.00±0.00	<0.0001
200	0.00±0.00	<0.0001	0.00±0.00	<0.0001	0.00±0.00	<0.0001

*Not related; a Untreated control

#### Cytotoxic effects

Different concentrations of DAS, MAT, and their mixture (in a 1:1 ratio) were studied on the macrophage cell line (Table 3). In the combination of drugs on amastigotes, the lowest IC50 value (38.34 μM) was observed compared to DAS (68.37±3.22) and MAT (83.22±4.26 μM) alone. The IC50 value of promastigotes (non-clinical stage) was meaningfully higher than that of intracellular amastigotes (*P*<0.001). The SI as the measure of the cytotoxicity level for three different treatments was obtained as 6.01, 10.53, and 15.21, respectively, which were found to be within the safety range (Table 3). The mixture of the two drugs was safer than each drug alone.

**Table 3:** Evaluation of IC50values for diallyl sulfide (DAS) and meglumine antimoniate (MAT) alone and their mixture (DAS+MAT) against promastigotes and amastigotes of *L*. *major* compared to meglumine antimoniate (MAT) and CC50 values of drugs on macrophages to calculate the selectivity index (SI).

Drugs	Amastigote		Promastigote		Macrophage	SI
	IC50 ± SD (μM)	P-value	IC50 ± SD (μM)	P-value	CC50 (μM)	
MAT	83.22±4.26	NR	267.37±38.33	NR	876.38±68.36	10.53
DAS	68.37±3.22	P<0.001	211.36±59.63	P<0.001	411.28±46.35	6.01
MAT + DAS	38.34±5.28	P<0.001	163.35±28.35	P<0.001	583.32±71.22	15.21

NR: Not related

#### Combination index (CI)

CI was used to predict the degree of drug interactions. Based on the equation the index was calculated to be additive (equal to 1) when the sum of the effects of the chemicals acting self-sufficiently (Fig 2B).

#### Th1 and Th2 related cytokines

**Fig 3:**
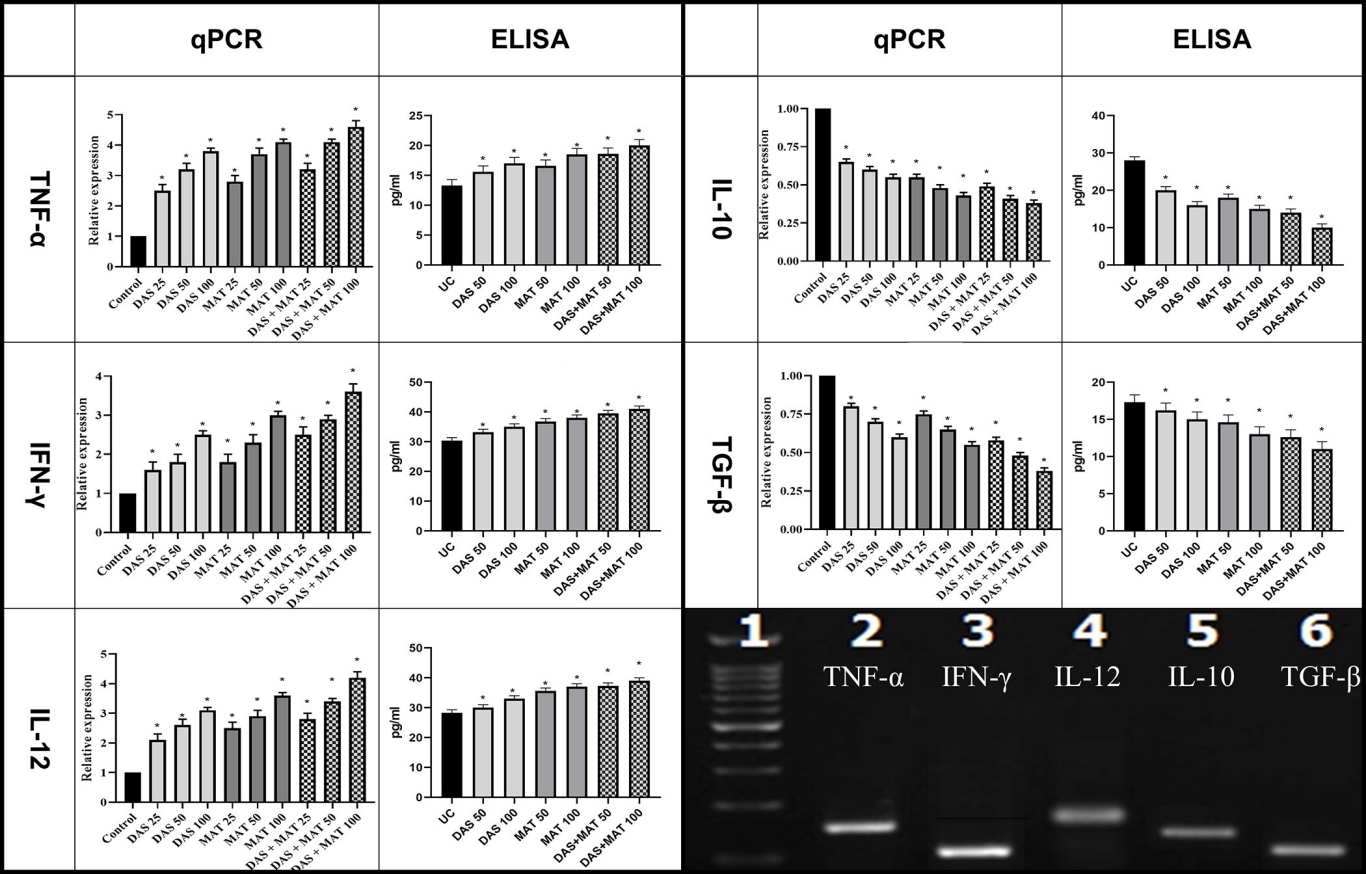
Th1 and Th2 the gene expression profiles and protein product in macrophages related to the relative expression of Th1-related cytokines including TNF-α, IFN-γ and IL-12, and Th2-related cytokines including TGF-β and IL-10 at different concentrations of diallyl sulfide (DAS), meglumine antimoniate (MAT) alone, and their mixture (DAS+MAT) in comparison to the control group. Bars are standard deviation (*P <0.001).

The expression of selected genes associated with Th1 and Th2 cytokines is presented in Fig 3, along with their corresponding protein products. In the DAS and MAT groups alone, the gene expression of Th1 cytokines was significantly higher compared to the control group (*P* < 0.001). Similarly, the expression levels of genes associated with Th1 and Th2 cytokines in the DAS group were comparable to those in the MAT group. However, in the combined DAS and MAT group, the effect was more pronounced, leading to an increased expression of Th1 cytokine genes (*P* < 0.001). In contrast, the expression of genes related to Th2 cytokines decreased as the concentration increased in all three groups (*P* < 0.001). Furthermore, when examined using ELISA, it was observed that the expression level of Th1-related cytokine proteins increased significantly compared to the untreated control group (*P* < 0.001). Conversely, there was a decrease in the production of Th2-related proteins compared to the untreated control group (*P* < 0.001) (Fig 3).

#### SOD, CAT, and NO

**Fig 4:**
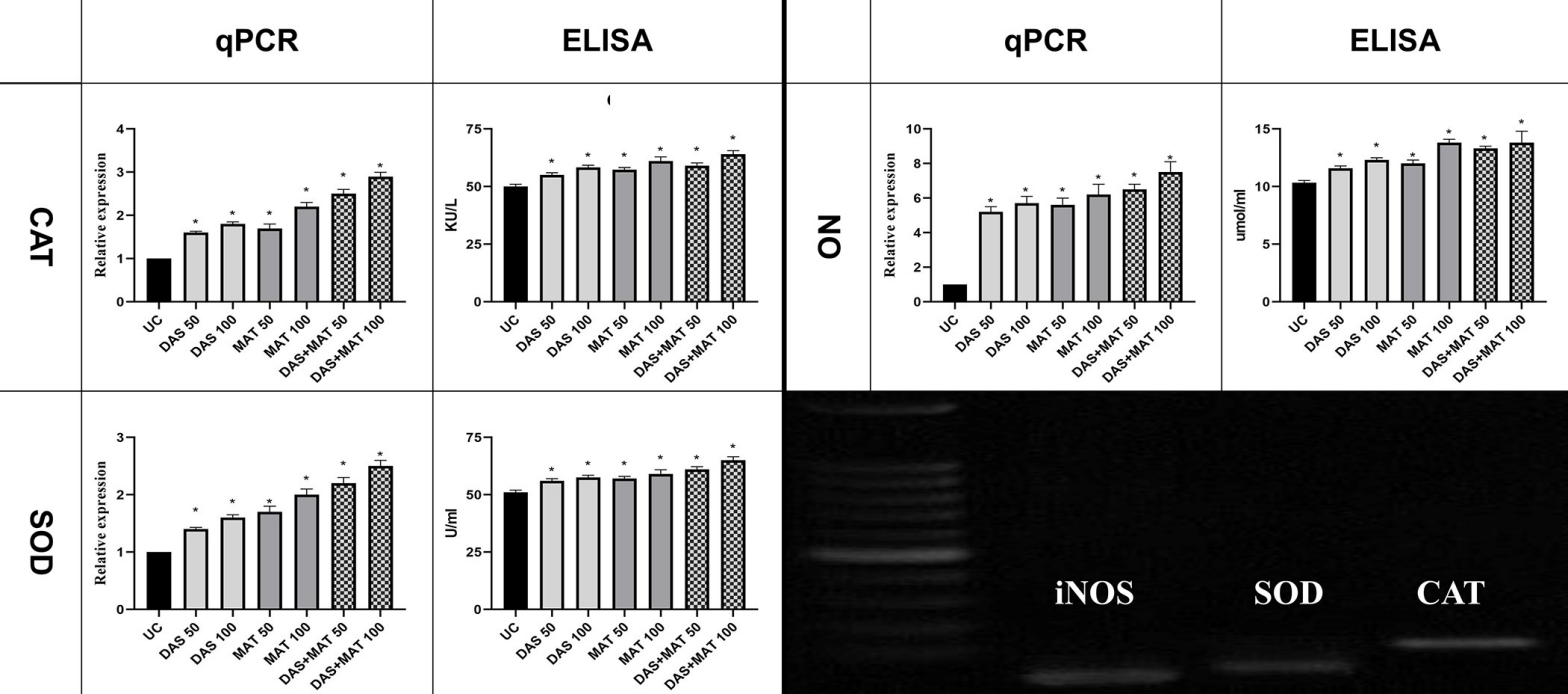
The effects of diallyl sulfide (DAS), meglumine antimoniate (MAT) alone, and their mixture (DAS+MAT) on intra-macrophages *L*. *major* amastigotes in comparison to the control group, on the level of gene expression and the activity of enzymes involved in the antioxidant index including superoxide dismutase (DAS), catalase (CAT) and nitric oxide (NO). Bars are standard deviation (*P <0.001).

The results obtained from qPCR and ELISA indicated a significant increase in SOD, CAT, and NO activity in the treated samples compared to the untreated control (*P* < 0.001). The observed increase followed a dose-dependent pattern and reached its highest level at a concentration of 100 μM. Notably, the combined treatment group exhibited higher activity values compared to the groups receiving DAS or MA alone (Fig 4).

#### ROS level changes in DAS, MAT, and their mixture

**Fig 5:**
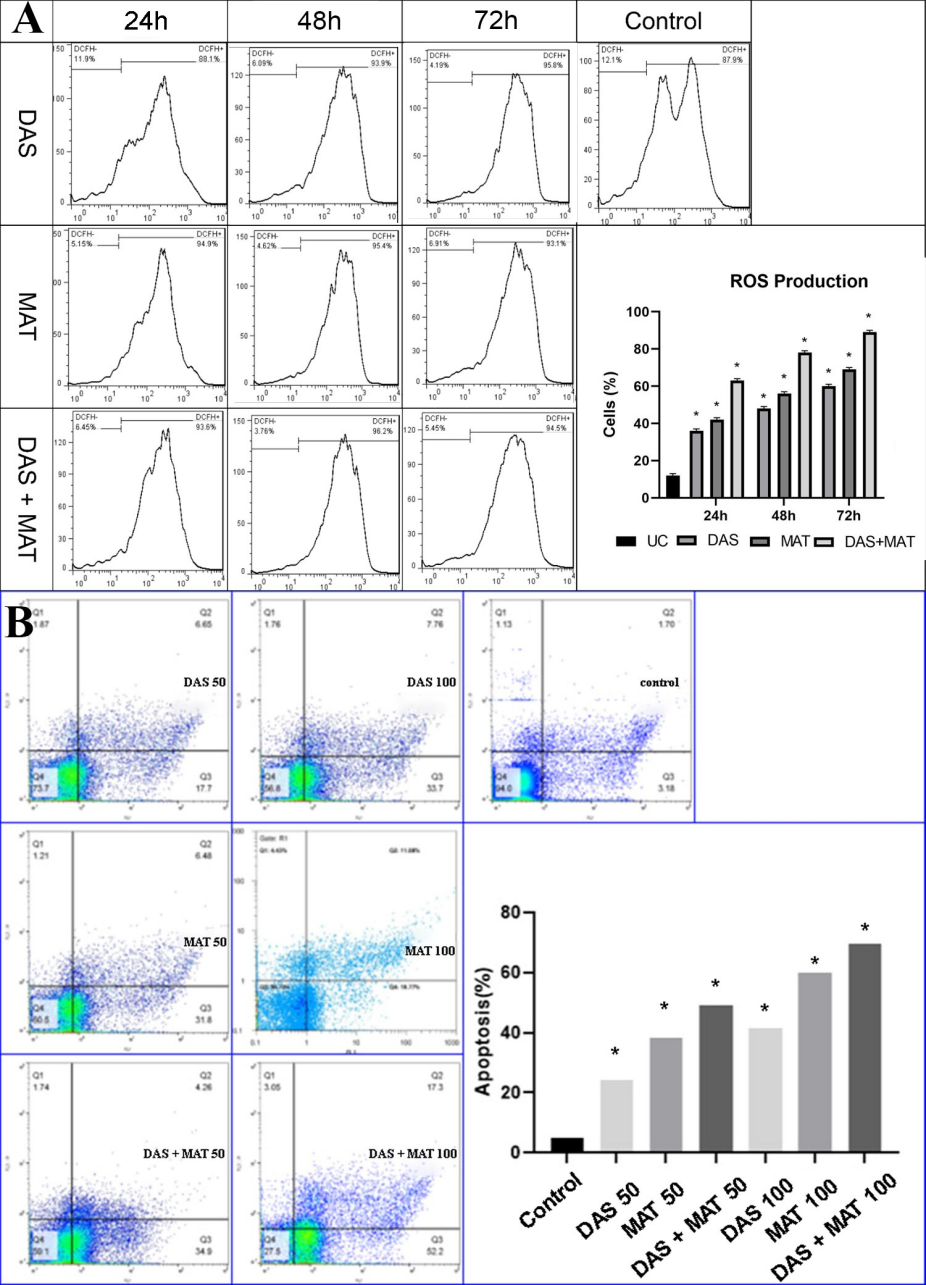
(A) The level of the cells that produced ROS at IC 50 concebtration of diallyl sulfide (DAS), and meglumine antimoniate (MAT) alone, and their mixture (DAS+MAT) in 24,48 and 72 hours (* significant difference with the untreated control group (UC) *P* <0.001). The effect of DAS was greater than that of MAT, while the effect of the mixture of the two drugs was greater than other modalities on the level of ROS production. (B) Illustrating the apoptotic profile of *L*. *major* promastigotes at different concentrations of diallyl sulfide (DAS), meglumine antimoniate (MAT), and their mixture (DAS+MAT) relative to the control group (*P <0.001).

IC50 concentrations of DAS, and MAT alone, and their combination promoted the extent of ROS production of amastigotes based on time-dependent reactions (Fig 5A).

#### Apoptotic profiles of DAS, MAT alone, and combination on L. major

Promastigotes treated with DAS, MAT, or their mixture significantly induced apoptosis. All three treatment schedules, at various concentrations, exhibited a substantial difference compared to the untreated control group (*P* < 0.001). It is worth mentioning that while MAT significantly increased apoptosis compared to DAS, the combination of DAS and MAT showed a significantly higher level of apoptosis compared to each drug alone or the untreated control group (*P* < 0.001) (Fig 5B).

#### TEM analysis

**Fig 6:**
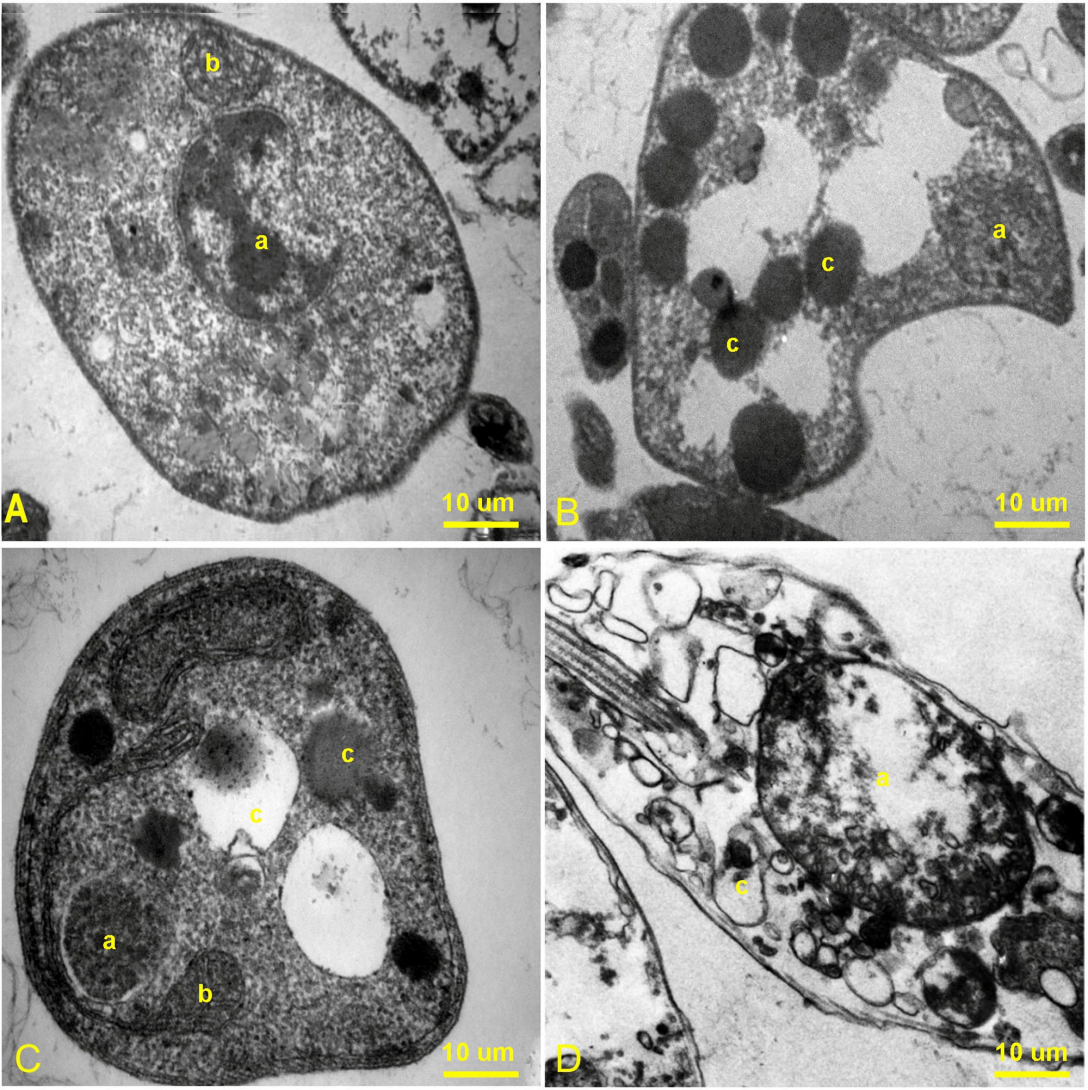
Ultrathin section of *L*. *major* promastigotes: A) untreated control group, B) MAT, C) DAS, and D) MAT + DAS. a) is the nucleus, b) is the mitochondrion and c) is the empty solid vacuoles.

TEM analysis showed several morphological and structural changes in treated promastigotes relative to the untreated control group. Structural alteration of mitochondria, the creation of vacuole formations in the cytoplasm, and changes in the assembly of the flagellum and other cell organelles were also observed. Most of these changes tended to be in the direction of creating the mechanism of apoptosis. While in the untreated control group, normal mitochondria and nuclei without any cytoplasmic and degenerative changes were seen (Fig 6A). In the DAS and MAT groups alone, some degenerative changes in mitochondrion and nucleus were observed, and many amorphous empty solid vesicles appeared (Fig 6B and 6C). In combination therapy, generalized and extensive degenerative changes, some signs of apoptosis in the nucleus and mitochondrion, and many empty spaces in the cytoplasm of promastigotes were detected (Fig 6D).

### C) *In vivo*

#### Different treatment schedules using DAS and MAT alone, and their mixture in infected mice

**Fig 7:**
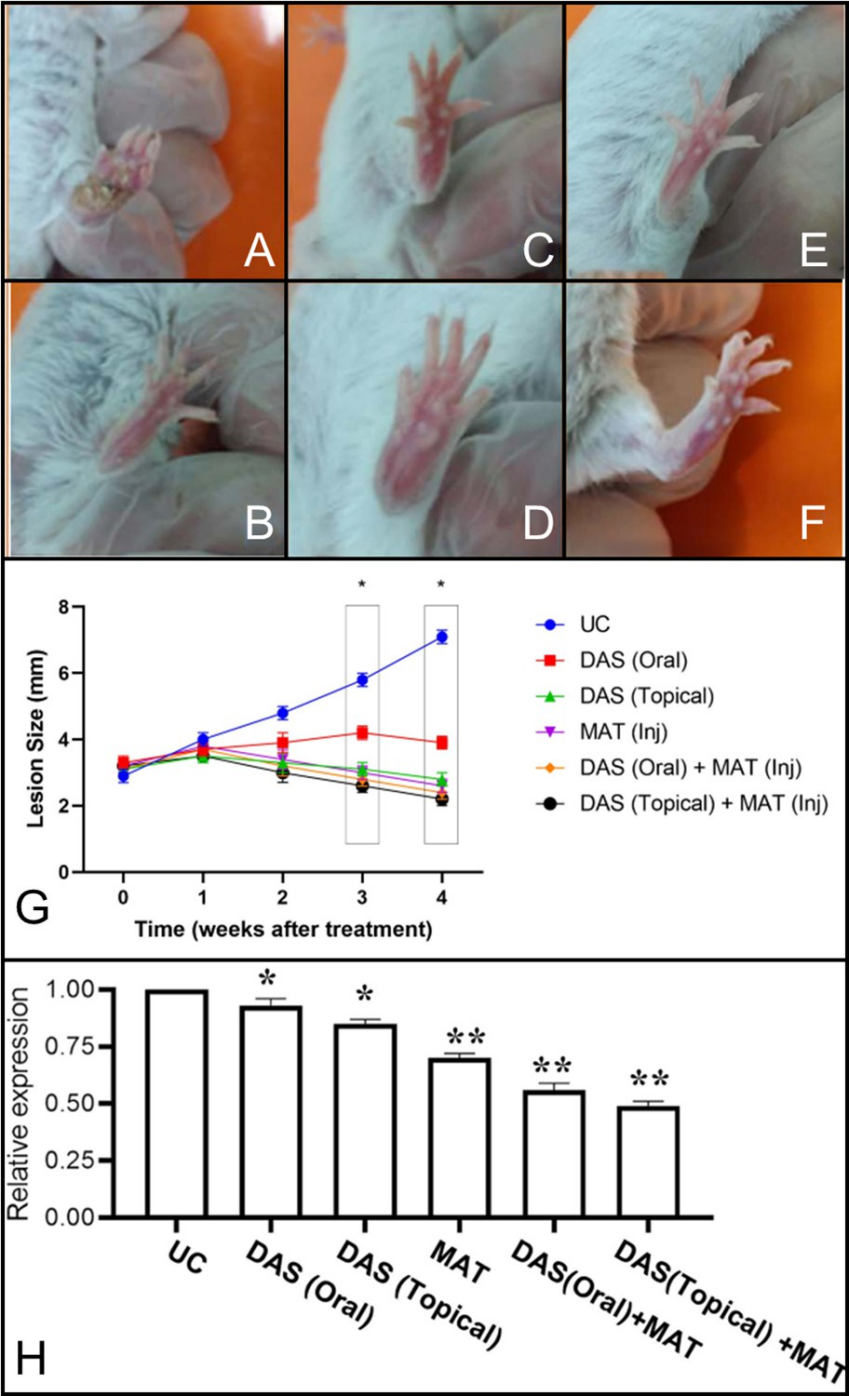
Changes in footpad lesion size in different groups of (A) UC (untreated control) (B) Meglumine antimoniate (MAT) (C) Diallyl sulfide (DAS) topical, (D) DAS oral, (E) (DAS oral + MAT), (F) (DAS topical + MAT) following the four weeks of treatment. (G) The cure rate in the left footpad is noticeable in the mixed group (DAS topical + MAT) compared to each group alone. The combination of the (DAS topical + MAT) group significantly reduced the footpad diameter compared to the other group (*P <0.001). **(H)** Gene expression changes in different groups of untreated control (UC), DAS oral and topical), MAT alone, and their mixture (DAS oral+ MAT), and (DAS topical + MAT) (*significant difference with UC *P* <0.05), (** significant difference with UC *P* <0.001).

The diameter of cutaneous lesion changes in different groups is shown in Fig 7A–7D. No detectable changes were observed in the untreated control group (healthy group) following the fourth week of the treatment regimen, while, in the untreated BALB/c mice, a gradual increase in the lesion diameter was detected and reached a maximum of 7 mm in diameter at the termination of the treatment schedule. In contrast, the lesion size significantly decreased in all groups (*P* <0.001) except DAS, which was orally received during the four weeks of treatment. Among different treatment modalities, combination therapy using DAS topical along with MAT achieved the best result in reducing the lesion size at the termination of treatment.

#### SODB1 gene expression profile in infected mice treated with DAS, MAT alone, and their mixture

Changes in SODB1 gene expression in the spleen of BALB/c mice treated by various approaches are shown in Fig 7H. The parasite load was significantly reduced in each treatment group relative to the untreated control group. At the same time, the least SODB1 gene expression was observed in the MAT plus DAS topical group, confirming the efficacy of this combination.

#### Histopathological and IHC changes

**Fig 8:**
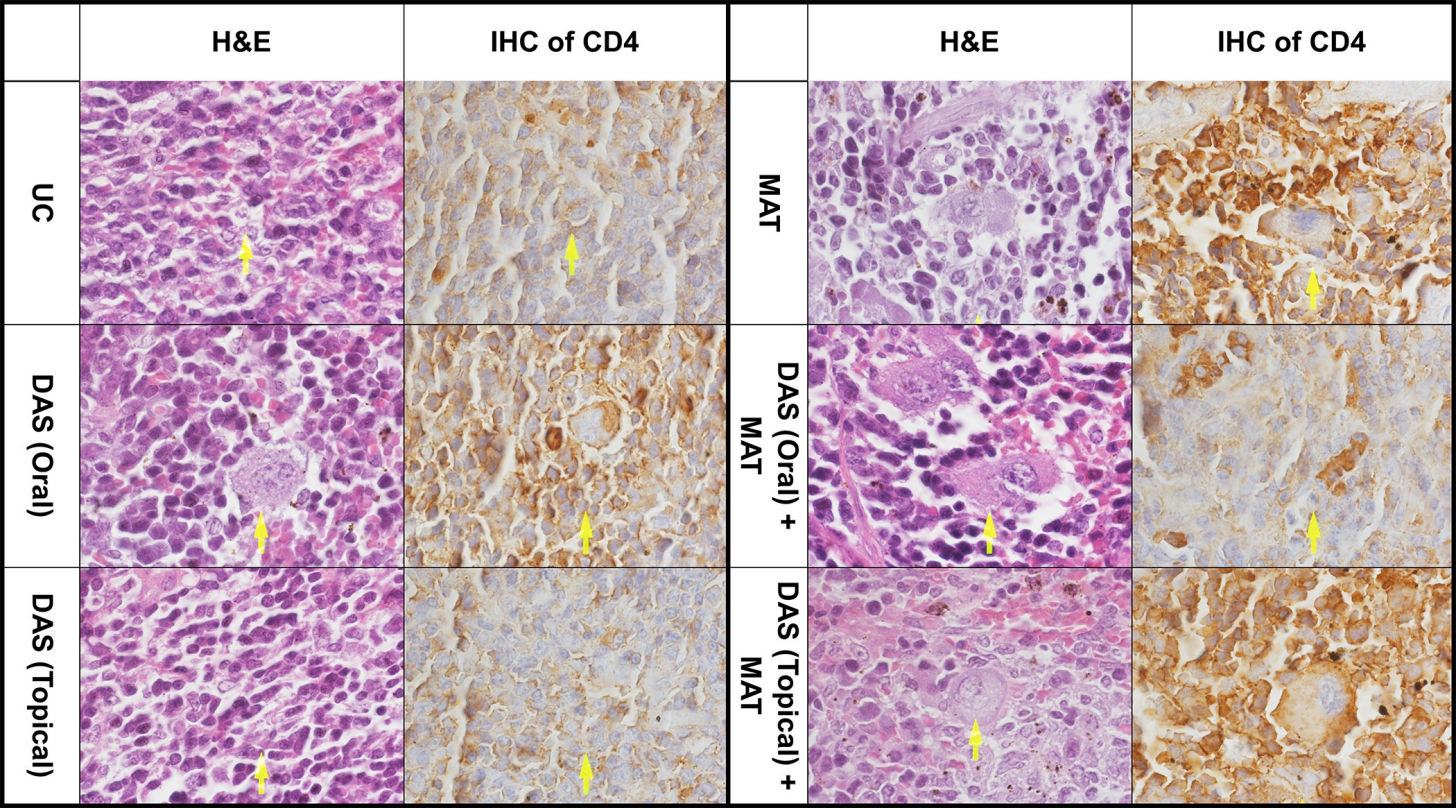
Histopathological (hemotoxin and eosin (H&E) stained) and immunohistochemical (CD4 antibody stained) changes of the spleen in *L*. *major*-infected BALB/c mice treated with different drugs including diallyl sulfide (DAS) in oral and topical form, meglumine antimoniate (MAT) and combination of them (DAS oral + MAT), (DAS topical + MAT) compared to the untreated control (UC) group.

**Fig 9:**
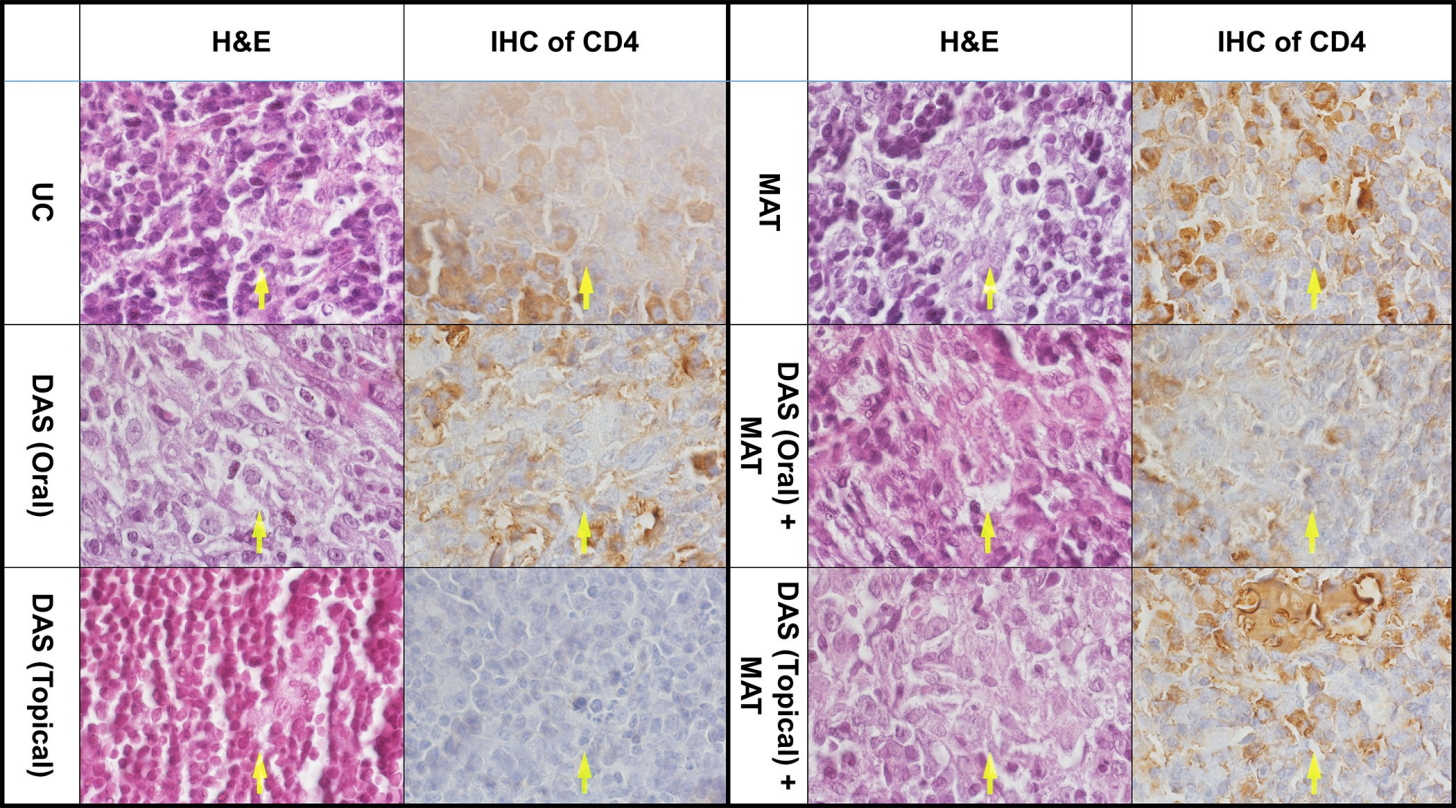
Histopathological (hemotoxin and eosin (H&E) stained) and immunohistochemical (CD4 antibody stained) changes of the lymph nodes in *L*. *major*-infected BALB/c mice treated with different drugs including diallyl sulfide (DAS) in oral and topical form, meglumine antimoniate (MAT) and combination of them (DAS oral + MAT), (DAS topical + MAT) compared to the untreated control (UC) group.

In histopathological analysis, the untreated control group demonstrated a collection of activated macrophages rimming by lymphocytes and inactivated macrophages in the spleen and lymph nodes (Figs 8 and 9; Table 4). The arrows show the presence of intracytoplasmic Leishman bodies (amastigotes). The DAS (oral) group indicates multinucleated giant histiocytic cells with intracytoplasmic Leishman bodies and empty parasitophorous vacuoles. Also, the DAS (topical) shows an assembly of activated macrophages containing Leishman bodies in the sinusoid and lymphocytes concentrated between them. The CD4 IHC result in DAS (oral and topical) groups showed increased macrophages compared to the untreated control group. MAT represents many activated macrophages engulfed-Leishman by bodies within the congested sinusoid, mixing with lymphocytes and a few plasma cells. The macrophages in this group showed empty vacuoles with degenerated Leishman bodies in parasitophorous vacuoles in the red pulp of the spleen and lymph nodes. CD4 IHC staining also confirmed increased lymphocytes around the damaged macrophages. DAS (oral) + MAT group engulfed Leishman bodies with activated macrophages that were heavily rimmed by lymphocytes, plasma cells, and inactivated macrophages. Still, compared to DAS (oral and topical) with the untreated control groups, the number of Leishman bodies in macrophages decreased so much that the CD4 IHC results confirmed an increased number of lymphocytes around the damaged macrophages. DAS (topical) + MAT group shows the presence of activated macrophages with intracytoplasmic containing few Leishman bodies sensitive cells were more hepatocytes than lymphocytes. Also, red pulp in the spleen and lymph nodes decreased compared to the untreated control group. CD4 marker in the IHC result confirmed an increase in the number around the macrophages in both tissues. Based on Ridley’s grading score, the best result was obtained when the infected mice were treated with DAS (topical) coupled with MAT compared to other treatment modalities (Table 4).

**Table 4:** Average parasite load according to the Ridley grading system quantified in 5 H&E stained popliteal lymph node sections corresponding to various groups of BALB/c mice including untreated control (UC), diallyl sulfide (DAS oral), meglumine antimoniate (MAT), diallyl sulfide (DAS topical), and their mixture (DAS oral + MAT) and (DAS topical + MAT).

Groups	No. of amastigotes/5 sections
UC	5,5,6,5,6
DAS (oral)	5,4,5,4,5
MAT	1,0,1,0,1
DAS (topical)	0,1,2,1,1
DAS (oral) + MAT	3,4,3,4,3
DAS (topical) + MAT	1,1,0,0,0

## Discussion

Immune-stimulating phytochemicals are the primary and logical leishmanicidal potential and provide novel strategies against leishmaniasis alone or in combination. Many of the macromolecules extracted from the plant origin show strong properties on the function of the immune system in experimental models, and their beneficial actions have been documented in many studies [31–33].

As an effective compound derived from garlic, Diallyl sulfide has been employed against many microbial and non-infectious diseases [34–37]. In this research, the influential inhibitory effects of DAS against *L*. *major* have been documented in experimental and animal models, notably when combined.

Selectivity index as a toxicity measure showed that DAS is relatively safe for mammalian macrophage cell lines as it was found to fall within the safety limits (SI=6.01). Similarly, the combination of MAT and DAS was much safer when the safety index (SI=15.21) was assessed.

Interestingly, all three treatment regimens (DAS, MAT, and DAS+MAT) represented a more potent effect on amastigotes than the promastigote stage. In other words, the clinical stage (amastigote) was more susceptible to medicines than the promastigote stage, although the clinical stage is intracellular [33, 38]. Therefore, pentavalent antimonial can be reduced to a trivalent form, the active ingredient substance at the amastigote stage. Besides, amastigotes easily concentrate therapeutic drugs rather than promastigotes, and this feature is mainly due to the difference in their biological and molecular functions [39].

The addition of DAS stimulates the acquired immunity under the influence of various cytokines. The main factors differentiating TCD4+ cells towards Th1 and stimulating those cells are IL-12, TNF-α, IFN-γ, and iNOS. Th1 cell-mediated immunologic response plays a vital role in managing *L*. *major*-associated CL. This phenomenon inspires the macrophages and kills surrounding amastigotes [23].

It has also been assumed that assessing differently expressed genes between dissimilar treatment groups may identify genes involved in treatment outcomes. Such immunomodulatory expressed genes can be useful as important targets for therapeutic screening and provide a coherent foundation for drug selection in CL. Even though removing *Leishmania* parasites by host immunity is highly complicated and multifactorial, innate susceptibility or resistance is closely related to well-defined subgroups of CD4+ T-helper lymphocytes [40, 41]. Cytokines have a crucial effect on the pathophysiology and host protection against leishmaniasis. In this study, it has been shown that DAS stimulates the gene expression of transduction trails and enables host cells to hamper the proliferative potential of *Leishmania*, thereby causing the death of the organism. The clinical cure or resistance to leishmaniasis contributes to Th1 propagation and the production of pro-inflammatory cytokines, including IL12p40, IFN-γ, and TNF-α, which leads to macrophage proliferation and eventual parasite death [42].

Mostly, phagocytic cells induce TNF-α, which could have a decisive effect on *Leishmania* death by creating NO synthesis in macrophages [41]. Besides, TNF-α increases the IFN-γ response against infection. Interestingly, TNF-α and IFN-γ induce macrophages to stimulate NO production by iNOS [41, 43, 44]. The present study shows that DAS promotes the expression of the iNOS gene and Th1-mediated cytokines in macrophages infected with amastigotes.

Many pieces of evidence show that several plant compounds have immunomodulatory potentials and thus can be a significant alternative or adjunct to standard chemotherapy in treating leishmaniasis [45]. Recently, comprehensive investigations have widely documented antileishmanial alternatives to drug therapy [46], but there is less evidence on how drugs work against amastigotes.

This study demonstrates that DAS effectively created interactions with the Th1 proliferation and pro-inflammatory cytokines. The foundation for molecular docking arrangements was the strength of binding of DAS and the four main immune system defense molecules iNOS, IFN-γ, IL-12, and TNF-α. Therefore, the relative free total energy of interactions of the diallyl disulfide with iNOS, IFN-γ, IL-12, and TNF-α implies that the DAS was significantly effective. Also, there is definitive approval that the binding affinity of DAS with iNOS, IFN-γ, IL-12, and TNF-α has been promising. Likewise, ADME and drug-likeness analyses revealed the appropriate drug-likeness possessions of the DAS, which indicates this compound can perform as a drug and offer significant biological processes. The findings of experimental analyses, molecular docking, ADME studies, and drug-likeness features are in suitable consensus, suggesting that DAS could be used as an effective treatment for *Leishmania* illnesses. The leishmanicidal effects of DAS and MAT are also mediated by apoptotic-like effects, as evidenced by the externalization of phosphatidylserine (PS) [23, 47]. This significant change appears due to the decrease in the activity of phospholipid translocase and the activation of calcium-dependent scramblase. The cytoplasmic bilayer’s equilibrium is disturbed during apoptotic processes, and PS is translocated to the cell’s outer layer [23]. The deposition of PS on the outer plasma membrane is a common appearance change between different apoptotic cells and cell cycle arrest at different growth phase stages. These findings indicate that the combination of DAS and MAT has a remarkable antileishmanial effect, presumably facilitated by a programmed cell death mechanism.

According to the definition, ROS arises from the metabolic activity of the cells and the production of highly active ion groups, molecules, and radicals. ROS appears in the mitochondria or as a product in the respiratory chain and contributes to many biological processes, such as hormonal biosynthesis, cell signaling, and the destruction of intracellular pathogens [48]. Also, ROS is an effective factor in dealing with intracellular pathogens that are encouraged primarily by IFN-γ and toll-like receptors [49]. In the life cycle, *Leishmania* parasites infect mammalian macrophages, where upon entry of the organisms, they produce significant quantities of ROS to halt the infection. This study’s findings align with the data described by others [50–53].

The mechanism of action of many leishmanicidal active constituents, including DAS and MAT, is in part by the stimulation of ROS production to promote the reduction process of the organism. Also, high amounts of ROS are a deadly weapon used by phagocytic cells to damage vital organic substances such as protein, lipids, and DNA and eventually lead to apoptosis [53, 54]. ROS has also been shown to be generated in leishmanial agents due to cellular and drug uptake. Various chemotherapeutic compounds used against *Leishmania* species and cancer treatment facilitate their effects by producing ROS [48, 50].

This investigation also displays that DAS significantly reduces the parasite burden of popliteal lymph nodes in infected mice within four weeks of injecting the treatment schedule. In addition, DAS (topical) + MAT compared to DAS (oral), DAS (topical), MAT, and DAS (oral) coupled with MAT pointed to a significantly lower parasite load in the skin lesions of BALB/c mice as measured by SODB1 gene expression.

Metacyclic promastigotes infect macrophages that are infiltrated in the lesion. Promastigotes transform and differentiate into amastigotes where they replicate inside these phagocytic cells. Macrophages will be activated through signal transduction by IFN-Ɣ and generate superoxide onion as radical species via complex biological processes. In phagosomes where amastigotes reside, oxygen superoxide may undergo degradation to form hydrogen peroxide or generate other ROS byproducts to inhibit parasite proliferation [56, 57].

*Leishmania* parasites also induce different types of interferon production by infected macrophages and, in turn, enhance the expression of SOD and CAT enzymes and act as potent antioxidants where they activate macrophages [55, 56] to produce proteolytic enzymes and ROS intermediates which contribute to the death of the organism. Mammalian host cells possess a broad range of antioxidant enzymes that consist of metal redistribution (iron, copper, selenium, and zinc) for their metabolism to protect against ROS. Superoxide dismutase and CAT remove harmful ROS from the milieu by catalyzing the dismutation of superoxide radicals to H2O2, H2O, and O2. The antioxidant enzymes act as a virulent factor in the macrophage and significantly decrease intracellular parasites [57].

The combination of DAS+MAT in topical form was superior, followed by DAS+MAT oral, DAS, and MAT alone. The study exhibited that DAS combined with MAT significantly reduced the amastigote load and lesion size in BALB/C mice, as confirmed by the grading scale of Ridley [24]. The composition of inflammatory immune cells, particularly the CD4+ sub-population, possibly affected the intracellular parasites. The IHC pathological marker was also another important tool for studying the pathogenesis of the disease. As the population of CD4^+^ T-lymphocytes increased, the number of Leishmanan bodies significantly decreased, as clearly represented, especially when the drugs were used in the mixture.

The present study confirmed that DAS has a proven anti-leishmanial activity. However, its lethal effect significantly increased when combined with MAT as the interaction between this mixture was an additive anti-leishmanial effect [58]. Such an effect frequently happens when two similar drugs accomplish an equal therapeutic outcome, although reducing the exact detrimental effect of one specific drug. The combination of herbal immunomodulators with standard drugs provides effective treatment of a variety of molecular targets, improving therapeutic efficacy and reducing toxicity [59]. Combination therapy, as emphasized in many studies, not only increases the effectiveness of CL treatment but also reduces the course and cost of treatment and diminishes adverse side effects, duration, and risk of parasite resistance [58, 60, 61].

In conclusion, in the present study, DAS represented a potent *in vitro* activity and a more enhanced lethal effect when combined with the standard drug (MAT). The mixture was safe and exhibited various mechanical actions, including promoting an immunomodulatory stimulation of Th1 cytokine phenotypes, apoptotic profile, ROS production, and antioxidant enzymes. This combination had a remarkable impact on lesion diameter, parasite load, and CD4+ marker exerted generalized degenerative structural changes at the subcellular level and improved the histopathological alterations in the murine model. Therefore, when used topically along with MAT, DAS deserves further advanced clinical trials and could be a potential candidate to treat volunteer cases with CL.

## Supporting information

S1 Raw images (PDF)

S1 Dataset (XLSX)
