# Supplementary figures and images for: Assessment of the antileishmanial activity of diallyl sulfide combined with meglumine antimoniate on Leishmania major: Molecular docking, in vitro, and animal model
Source: PLoS One. 2024 Aug 30;19(8):e0307537. doi: 10.1371/journal.pone.0307537 (PMC11364230; doi:10.1371/journal.pone.0307537)

Related to figure 3

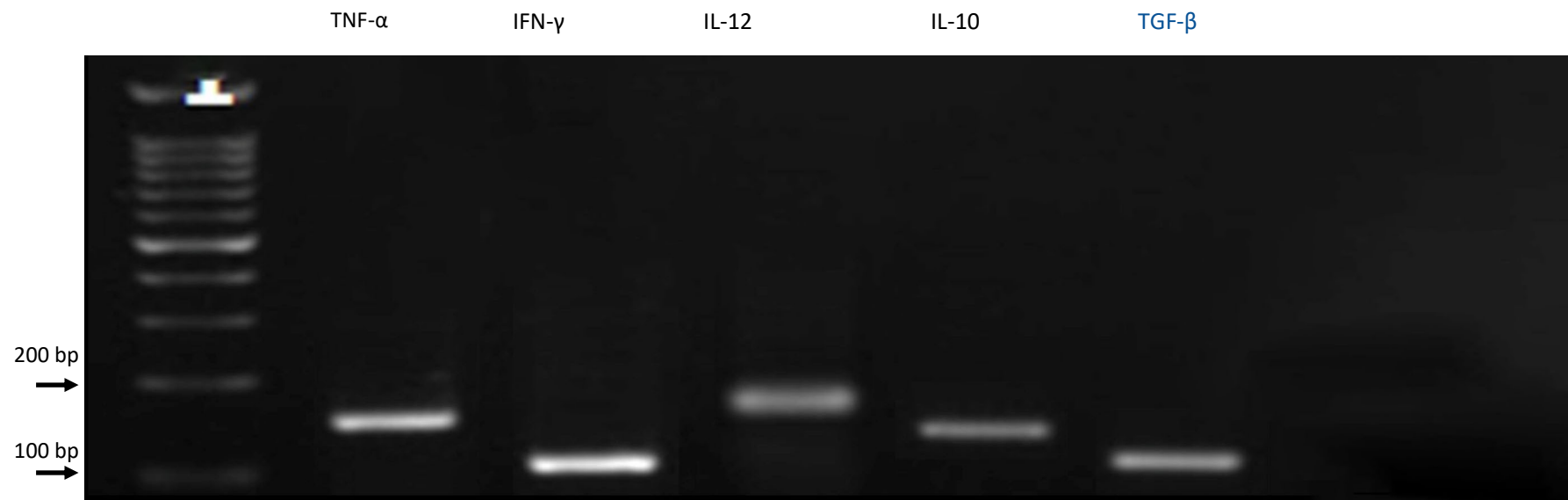

Related to figure 4

iNOS

SOD

CAT

200 bp  
→  
100 bp  
→

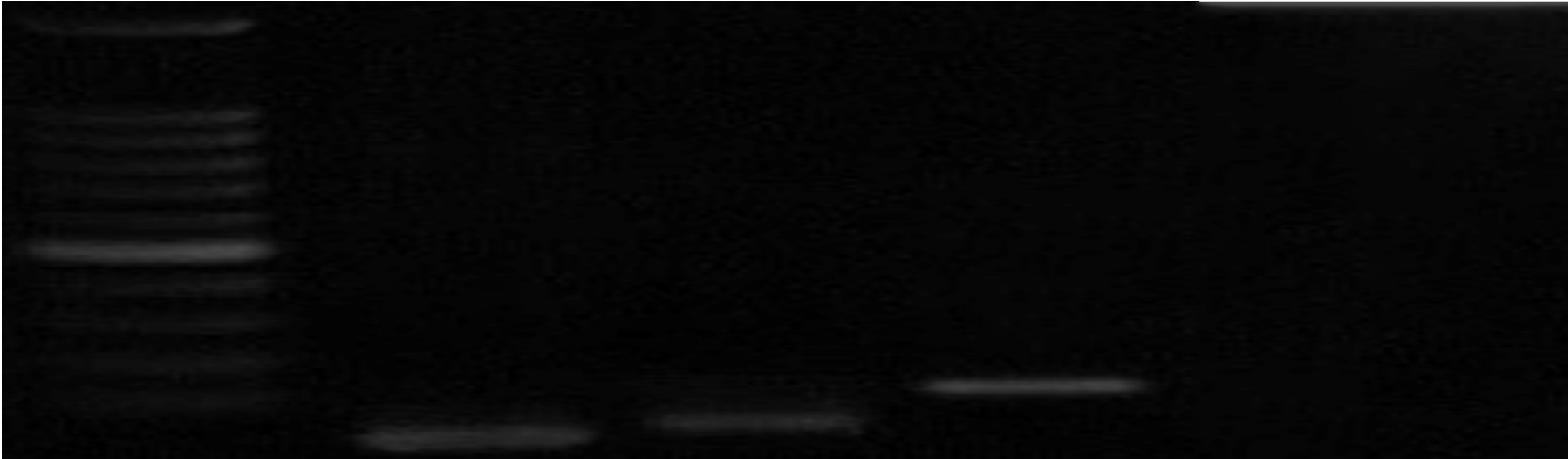

Supplement: S1 Raw images — (PDF) [file pone.0307537.s001.pdf]
